# Clinical and radiographic evaluation of melatonin and chitosan loaded nanoparticles in the treatment of periodontal intra-bony defects: A Randomized controlled clinical trial

**DOI:** 10.1007/s00784-025-06323-3

**Published:** 2025-05-02

**Authors:** Amira AL-agooz, Fatma Ata, Wafaa Saleh, Samah Elmeadawy

**Affiliations:** Oral Medicine, Periodontology, Diagnosis and Oral Radiology Department, Faculty of Dentistry, Mansoura University, Mansoura, 33516 Egypt

**Keywords:** Melatonin, Local drug delivery, Periodontal defects, Nanoparticles, Periodontitis

## Abstract

**Objectives:**

The current literature lacks the effect of melatonin loaded nanoparticles (LNPs) as local drug delivery (LDD) in the treatment of periodontitis. Hence, the aim of the current study is to investigate the clinical and radiographic effects of melatonin LNPs in patients with periodontal intrabony defects.

**Methods:**

The current study was performed on healthy patients with periodontal intrabony defects. The participants were randomly allocated into 3 groups. Group 1 received scaling and root planing (SRP) with melatonin LNPs, group 2 received placebo gel with SRP, and group 3 received SRP and chitosan LNPs. The primary outcomes included the radiographic measurements of the bone defects to evaluate the bone fill after 6 months. The secondary outcomes included the following clinical parameters; clinical attachment level (CAL), periodontal probing depth (PPD), plaque index (PI), and gingival index (GI). The clinical parameters were evaluated at baseline, 3 months, and 6 months.

**Results:**

The current study included 67 patients with periodontal intrabony defects. All the study groups demonstrated significant improvements in all the clinical outcomes (CAL, PPD, PI, and GI) (P < 0.05). Melatonin LNPs group revealed the most significant improvement of the radiographic outcomes after 6 months including bone defect height and depth, alveolar crest level, and the buccolingual and mesiodistal width of bone defects) (P < 0.05), followed by chitosan group while insignificant changes were detected in the placebo group (P > 0.05).

**Conclusion:**

Melatonin LNPs as a LDD can act as a promising therapeutic modality in treating periodontal intrabony defects through significant improvement of the clinical and radiographic outcomes.

## Introduction

Periodontitis is a chronic inflammatory disease characterized by destruction of tooth-supporting tissues [1]. Untreated periodontal diseases may result in formation of intra-bony defects and eventual tooth loss [2]. Progression of chronic periodontitis is characterized by increased osteoclast activity, which leads to formation of intraosseous defects [3]. Management of periodontal disease involves a range of evidence-based treatment modalities designed to control infection, arrest the progression of tissue destruction, and restore periodontal health. [4–8]

Adjunctive antimicrobial agents are crucial for patients with persistent periodontal deterioration despite regular mechanical treatments [9]. The delivery method and dosage form have a beneficial effect on the therapy's overall clinical result. [10]

Local drug delivery (LDD) in periodontal diseases aims to enhance the therapeutic profile of the drug and reduce side effects. The therapeutic goal of LDDs is met by directly injecting antimicrobial agents into the periodontal pocket and subgingival sites. This results in active release of the medication in a controlled, sustained manner to fight the microbial attack while minimizing the unfavorable effects [11, 12].

Compared to systemic antimicrobial agents, LDDs offer a number of benefits, such as they increase the concentration of the therapeutic substance in a specific targeted area to achieve high concentration levels, decreasing systemic levels and achieving a reduction or elimination of the possible adverse effects in other tissues [13]. LDDs can be obtained as irrigating systems, fibers,gels, strips, films, microparticles, and nanoparticle. [14]

Melatonin is a hormone secreted by the pineal gland, retina, bone marrow, and immune system. Its primary function is circadian rhythm regulation [15]. Melatonin has been shown to influence bone metabolism[16, 17], exhibiting anti-inflammatory properties and playing a potential role in enhancing bone formation [18–21]. It has been demonstrated that melatonin promotes bone repair around titanium dental implants. Because of its regulatory effects on inflammation, antioxidant capabilities, bone cell regulation, and synthesis of collagen. Melatonin may be essential at all stages of bone regeneration. Applying topical melatonin to the ostectomy site after implant placement has been demonstrated to enhance bone mass, density, and bone-to-implant contact, especially in the early stages of healing [22].

Chitosan has excellent performance as a biodegradable scaffold in tissue engineering due to its biocompatibility and functional flexibility [23, 24]. A variety of chitosan scaffolds, including microspheres and nanoparticles(NPs), have been created and engineered with the ability to regulate the release of growth factors obtained from the integrated scaffold [25–27].

Recently, nanotechnology has been introduced into the drug delivery field due to its unique features including the surface to mass ratio. [28] In medicine, nanotechnology has emerged as a transformative tool offering new avenues for enhancing drug delivery and tissue engineering. NPs are especially useful in periodontal therapy because they can encapsulate therapeutic substances, shield them from deterioration, and release them at the target region in a regulated and sustained manner [29]. Because of targeted delivery minimizes systemic adverse effects while maximizing the local therapeutic effects, making NPs an effective tool for periodontal regeneration [28].

In recent years, the use of loaded nanoparticles (LNPs) has gained attention from clinicians and researchers in periodontal regenerative surgery due to its effective drug delivery to targeted sites of infection and ability to enhance tissue regeneration. Both in vitro and in vivo studies have demonstrated the effective role of LNPs in improving periodontal outcomes [29]. Nanomaterials can deliver therapeutic agents to periodontal tissues effectively, enabling periodontal ligament and alveolar bone regeneration. A particular in vitro study has shown that nanofiber scaffolds containing nano-hydroxyapatite can promote healing of the periodontal wound by increasing the production of osteopontin and vascular endothelial growth factor which help in formation of new blood vessels [30]. The nanoparticles enhance the in vivo efficacy of bioactive molecules by helping them penetrate tissue easily, release drugs slowly and efficiently over time and achieve targeted delivery for better periodontal healing and regeneration [31]. In vivo research provides additional evidence that loaded nanoparticles helps to regenerate periodontal tissues [32]. In addition, studies on animals have shown that nanoparticles improved tissue regeneration by enhancing the cellular absorption of nanoparticles more efficiently, regulating the removal of damaged cells and enhancing new bone cells formation. For example, in rat models, the treatment with self-assembling peptide nanoparticles helped periodontal defects repair by increase functional PDL length and decrease the epithelial down growth [33]. In addition, administration of curcumin-loaded nanoparticles prevented inflammation and bone loss in experimental periodontal disease [34]. Another research on curcumin-loaded nanoparticles has shown that they enhance repair and promote healing of periodontal tissues after treatment in experimental models.[35] In a recent study, administration of 1% chitosan nanoparticle gel adjunct to SRP in chronic periodontitis patients resulted in a significant improvement in the clinical parameters while showing inhibitory action against the periodontal pathogens P.ginigivalis and T.forsythia [36].

In periodontal therapy, traditional local drug delivery (LDD) systems usually face some challenges including rapid breakdown in oral environment which reduce the amount of drug available at target site, resulting in less effectiveness, low solubility, limited in vivo stability, inefficient absorption, and difficulties in achieving prolonged and targeted delivery to site of action. In addition, LDD doesnt reach effective concentration in the periodontal pocket. Consequently, researchers have introduced nanosized drug delivery systems like (LNPs) which protect the drug from breakdown rapidly and enhancing stability and deep penetration into pockets. Additionally, LNP enable controlled, sustained release of the drugs in periodontal treatments with polymeric nanoparticles through enhancing the ability of penetration of the junctional epithelium and slow release of the drug and over a long time. Recent advances in nanotechnology have introduced new types of nanoparticles such as liposomes, metallic nanoparticles and polymeric meicelles [37–42].

This randomized controlled clinical trial aims to evaluate the clinical and radiographic outcomes of utilizing melatonin loaded nanoparticles (LNP)-based delivery system for the treatment of periodontal intra-bony defects. This study seeks to explore a novel therapeutic strategy to enhance periodontal regeneration and improve patient outcomes.

## Materials and methods

### Study design and population

The current study was designed as a triple-blind randomized controlled clinical trial including eligible participants with at least one intrabony defect that was diagnosed clinically and radiographically. The patients were selected from the Periodontology Clinic at the Department of Oral Medicine and Periodontology, Faculty of Dentistry, Mansoura University, Egypt. After discussing the study’s protocol, participants were required to sign an informed consent form to participate in the study and to adhere to the research schedule and protocol. This study was approved by the institutional ethics committee at the Faculty of Dentistry, Mansoura University, ethical approval number “A06011122” and it was registered in ClinicalTrials.gov under the unique identifier [NCT05906563].

We applied the following inclusion and exclusion criteria during selection of the study’s participants:

We included healthy participants with at least one maxillary or mandibular intrabony defect which have three walled or combined defects without involving the furcation were included in the study by an independent blinded investigator and were between the ages of 25 and 55 years old. The study included both single-rooted and multi-rooted teeth with intrabony defects**.** Participants were excluded if they had a history of known systemic diseases that affect the periodontal treatment, allergies, or use of chemotherapeutic agents; received antibiotics or periodontal therapy within the previous three months preceding our study; patients with furcation involvement, smokers or tobacco chewers; taking systemic drugs that affect metabolic bone diseases, had poor oral hygiene; or if they were pregnant females.

## Sample size calculation

The sample size of the current study was calculated based on the change in the mean clinical attachment level(CAL) between the study and control groups of previous research conducted by Chitsazi et al. [43]. Using G*power version 3.0.10 to calculate sample size based on the effect size of 1.49, 2-tailed test, α error = 0.05, and power 90.0% the total sample size was 20 periodontal intrabony defects in each group, one defect per participant.

## Randomization

Before starting the study, the participants were randomly allocated into three groups using a computer-generated system utilizing Excel 2013 v 15.0 for Microsoft windows [44, 45]. The participants were allocated to either one of the three groups by a co-investigator who was blinded to the study procedures and assessment of the study’s outcomes. To ensure blinding, the topical gels were prepacked and labeled with unique codes by a separate investigator while the codes were not disclosed to the participants, the principal investigator who conducted the clinical procedures, nor the statistician analyzing the de-identified data.

The study groups were categorized as follows: Group 1 (Melatonin LNP group) which included 23 Patients treated with melatonin LNPs gel as an adjunct to scaling and root planing(SRP); group 2 (placebo group) included 24 patients who were treated with placebo gel as an adjunct to SRP; group 3 (chitosan LNPs) which included 20 patients treated with chitosan LNPs gel as an adjunct to SRP.

### Primary and secondary outcomes

For each patient, we selected one defect prioritizing the deepest intrabony defect. In case of multiple intrabony defects with the same clinical and radiographic measurements, we performed randomization using a computer-generated randomization list to select the defect for treatment. We believe that the difference between several bony defect characteristics (depth and height) in the same patient could confound the results of the current study. The study’s outcomes were categorized into primary and secondary outcome:

## Primary outcome variables

The primary outcomes included the radiographic measurements of the bone defects to evaluate the bone fill and bone volume using cone beam computed tomography (CBCT). They were measured at the baseline as well as after 6 months from the start of the study.

The radiographic measurements included bone defect height, alveolar crest level, bone defect depth, and the buccolingual (BL) and mesiodistal (MD) width of bone defects. Imaging was performed using the SCANORA® 3D CBCT system (manufactured by SOREDEX, Tuusula, Finland) and operated at an adjustable tube voltage of 85–90 kVp and a tube current of 3–15 mA. The field of view (FOV) used in CBCT was small-to-medium plus field of view, specifically small S FOV was (50 × 50) mm, medium M (80 × 100) mm, and medium M + (80 × 165) mm, to minimize radiation exposure and provide details of the intraosseous defect.

**Fig. 1:**
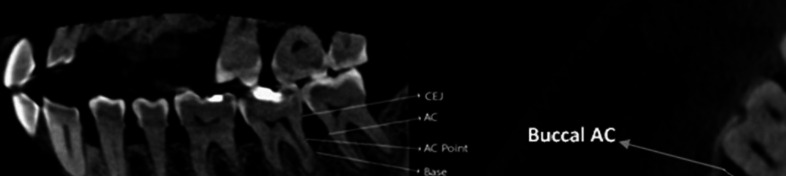
CBCT showing the radiographic measurement of the bone defect: (A) Sagittal view of CBCT showing the reference points; CEJ, base of the defect, alveolar crest (AC), and AC point.(B) Axial view of CBCT showing the BL width of the defect was measured in the axial plane as the horizontal distance between the most coronal point for the buccal and lingual ACs

Measurement of the bone defect height was made from the CEJ to the base of the defect (BD). The depth of the bone defect was measured from the BD to the alveolar crest (AC). The level of the alveolar crest was measured from cementoenamel junction (CEJ) to AC. The defect's BL width in the axial plane was measured as the horizontal distance between the buccal and lingual alveolar crest's most coronal points. The AC point was measured as the junction point of a line drawn perpendicularly from the AC to the root surface. The depth of the intraosseous defect was defined as the distance between AC point and the base of the defect (AC‒BD). The MD width of the intraosseous defect was defined as the distance between the AC point and the AC [46].(Fig. 1).

## Secondary outcomes variables

The secondary outcomes included clinical parameters such as CAL, periodontal probing depth (PPD), plaque index (PI), and gingival index (GI). The PPD was measured from the free gingival margin to the pocket base [47] while CAL was measured from CEJ to the base of the pocket with a UNC #15 periodontal probe [48]. The GI [49] comprises examination of all teeth surfaces, including the buccal, mesial, lingual, and distal surfaces. The score ranges from 0 to 3. The GI of an individual was obtained by summing the values determined for each tooth and calculating the averages. The PI determines the thickness of plaque along the gingival margin using a periodontal probe. Air was employed to dry the teeth for plaque visualization, which was not pigmented or stained. [50]

## Synthesis of the nanoparticles LDD:

### Materials and Reagents:

• Chitosan: Dissolved in 0.275 N HCl to yield a 46 mg/mL solution.

• Phospholipon 90G (Lipoid AG, Switzerland): 23 mg.

• Melatonin: 23 mg.

• Solvent: Absolute ethyl alcohol (2 mL).

• Water: Deionized, used as a dispersion medium.

##  Equipment Specifications

- Magnetic Stirrer: Used for mixing at a controlled speed of 600 rpm.
- Syringe (0.75 mm inner diameter): Used for injecting ethanol solution.
- Dynamic Light Scattering (DLS) (Zetasizer Nano ZN, Malvern Panalytical, UK): Used for particle size and zeta potential analysis at a fixed angle of 173° at 25 °C.
- Differential Scanning Calorimeter (DSC): Used to analyze thermal stability and crystallinity of nanoparticles. (Simultaneous Thermogravimetric Analyzers (STA): NEXTA STA200. Hitachi. Jaban).
- Spectrophotometer (JENWAY 6305, UK): Used for encapsulation efficiency measurement at 279 nm.
- Fourier Transform Infrared Spectroscopy (FTIR): [4000–500 cm]

## Synthesis Procedure:

Preparation of Drug-Loaded Nanoparticles:

To prepare the Melatonin LNPs, chitosan was dissolved in 0.275 N HCl to yield a concentration of 46 mg/mL. Then, 500 μL of this solution was added to 20 mL of water, while the pH was adjusted to 3 and the volume was 23 mL to obtain a final concentration of chitosan 1 mg/ml. 23 mg of Phospholipon 90G (Lipoid AG, Swizerland) and 23 mg of melatonin were dissolved in 2 ml of absolute ethyl alcohol. Melatonin LNPs were formed spontaneously by injecting 2 mL of the ethanol solution of phospholipon and melatonin into 23 mL of the chitosan solution under stirring. For an hour, the stirring continued until the complete evaporation of ethanol. The nanoparticle was prepared using a 1:1 phospholipon-to-chitosan ratio. The chitosan LNPs were formed using the same technique without adding melatonin. The placebo gel included empty NPs which were prepared in the same manner, without adding melatonin or chitosan.

- **Characterization and Validation:**
- Particle size and zeta potential were determined using DLS.
- Drug encapsulation efficiency was calculated using UV–Vis spectrophotometry at [279 nm].
- Chemical stability and crystallinity were assessed via FTIR.
- **Standard operating procedures for drug storage and stability maintenance**

1. Temperature: Samples were stored at 4 °C in an airtight container to prevent degradation.

2. Humidity control: Maintained in a desiccator to prevent moisture absorption.

3. Light protection**:** Stored in amber vials to minimize light-induced degradation.

## Stability maintenance

Short-term stability: Evaluated over one month at 4 °C, measuring particle size, zeta potential, and polydispersity index (PDI).

## Characterization of nanoparticles:

### Determination of the entrapment efficiency of melatonin

**Fig. 2:**
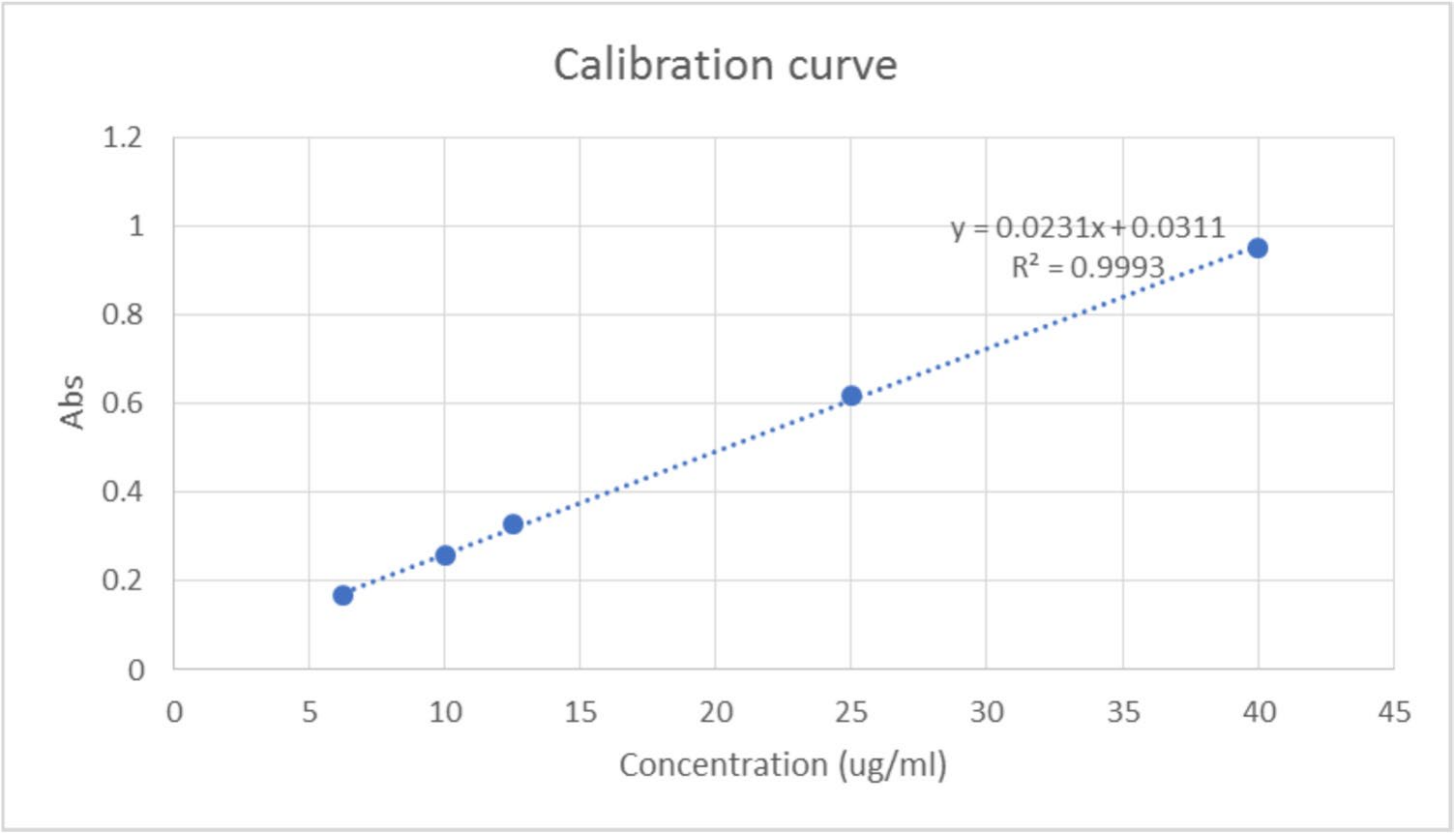
Standard Curve to Measure Concentration of Free Drug(melatonin) [52]

Three independent samples of the provided formula were tested (0.5 ml was tested for each sample). Each sample was added to a centrifugal filter unit (Amicron Ultra − 10 K, Merk Millipore, Tullagreen, Carrigtwohill) and centrifuged at 3000 g for 40 min at 4 °C. The supernatants were collected for calculation of their drug content, which corresponds, to the un-entrapped drug (indirect method). Drug concentration was measured using a JENWAY 6305 UV Spectrophotometer (Staffordshire, UK) at wavelength = 279 nm. The chitosan nanoparticles filtrate was used as blank A standard curve was created using several serial dilutions (Fig. 2). For each sample, the three recorded readings for triplicate samples of Melatonin LNPs were averaged. The following equation was used to calculate entrapment efficiency(EE). [51]:(1).

EE% = Total Melatonin − Free Melatonin/Total Melatonin × 100.

## Measurement of particle size

After preparation of the nanoparticles, the samples were diluted tenfold with deionized water and reanalyzed. Using photon correlation spectroscopy and a particle size analyzer (Dynamic Light Scattering(DLS), Zetasizer Nano ZN, Malvern Panalytical Ltd, UK), the prepared particles were measured for size and distribution in terms of average volume diameters and polydispersity index at a fixed angle of 173° and 25 °C. For maximum reliability, each sample was examined three times. The particles'zeta potential was also measured using the same device.

### Particle size, polydispersity index, and zeta potential measurements

**Fig. 3:**
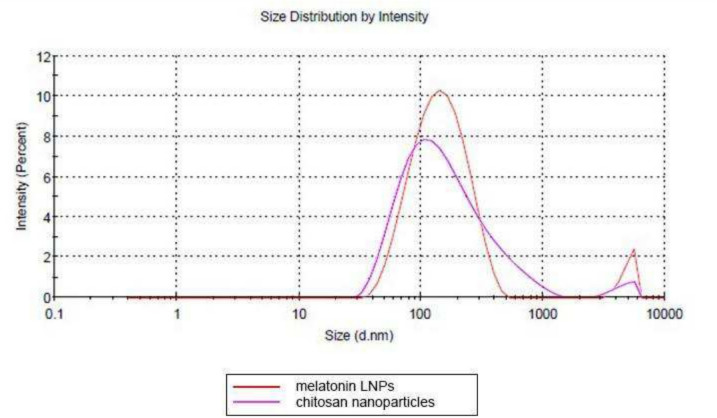
Characterization of nanoparticles: Dynamic Light scattering measurement of mean hydrodynamic size of melatonin LNPs and chitosan nanoparticles

The EE of the melatonin within nanoparticles was 11.30%. The chitosan LNP had an average size of 194.8 ± 9.5 nm and the melatonin LNP had an average of 161.6 ± 10.33 nm. Despite their increased size, chitosan LNPs did not differ significantly from melatonin LNP. (Fig. 3).

Polydispersity index (PDI) is an indicator for the homogeneity of particles size within a formulation and it is calculated based on the sample’s particle size distribution. PDI was 0.544 ± 0.107and 0.375 ± 0.102 for chitosan-LNP and Melatonin LNP, respectively. The zeta potential presented positive values for both NP preparations and there was no statistical difference between the values.

**Fig. 4:**
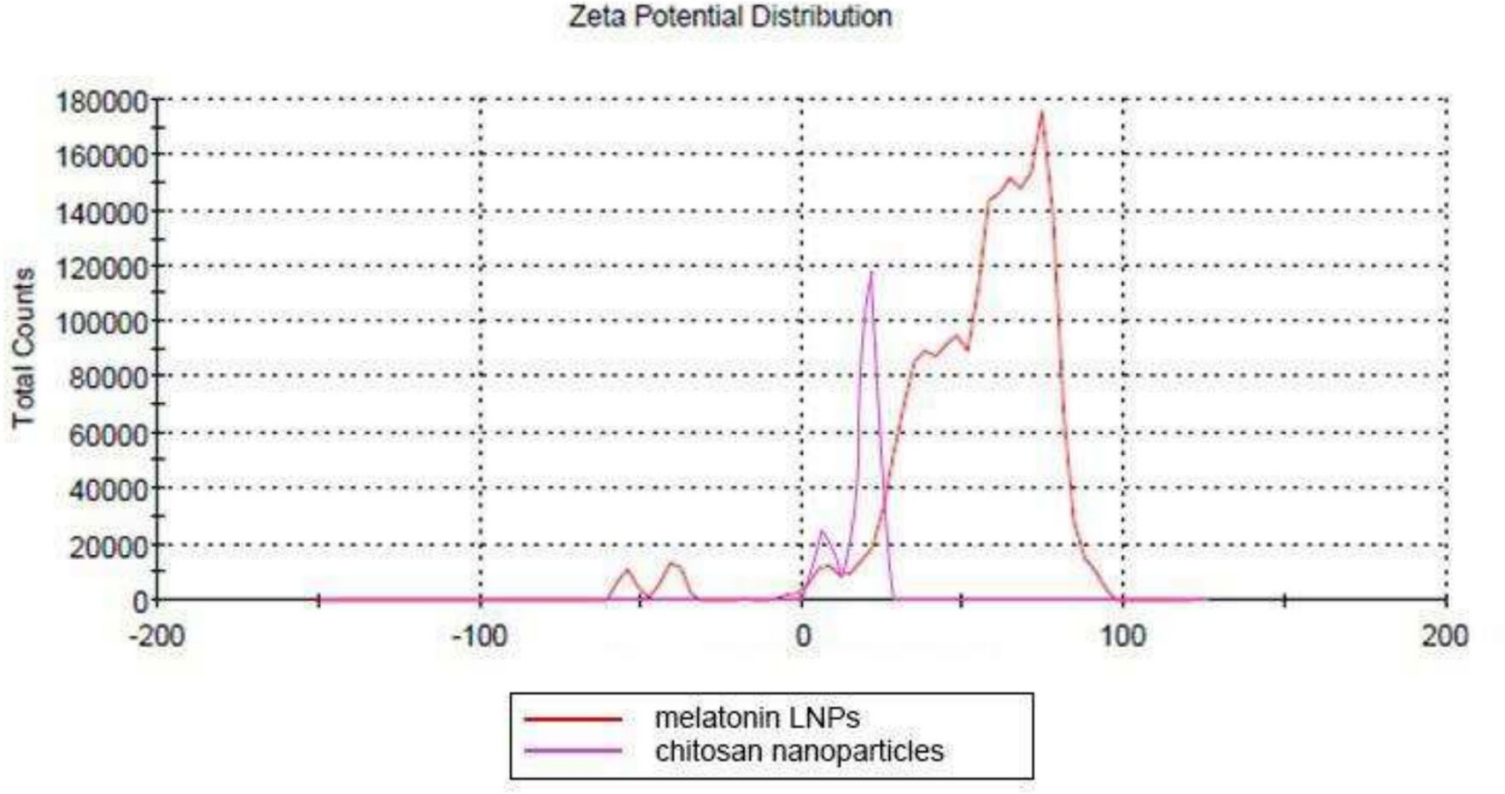
Surface zeta potential measurement of melatonin LNPs (53.7 mV) and chitosan nanoparticles (18.9 mV)

In the current study, zeta potential values of all prepared formulations ranged over 52.9 ± 0.907 which indicate good stability of the system. And 19.9 ± 3.76 for chitosan nanoparticles. (Fig. 4).

## Transmission electron microscopy (TEM)

**Fig. 5:**
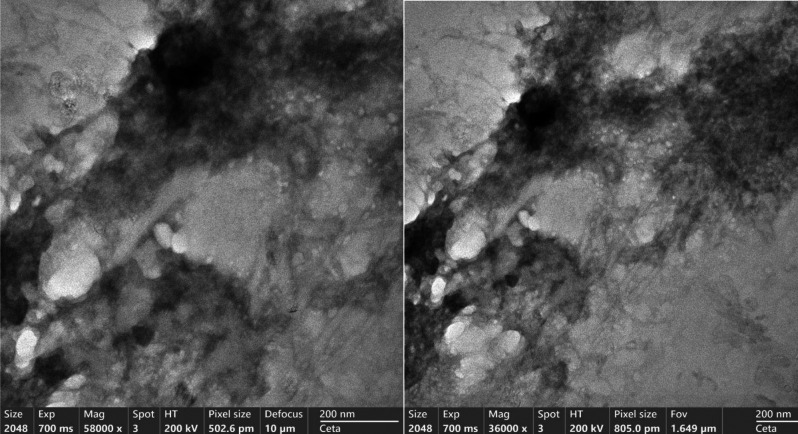
TEM images of Melatonin LNPs show nano-sized particles

The particles morphology and surface characteristics were examined by TEM (JEOL 2100; JEOL, Tokyo, Japan). For the sample preparation, one drop of freshly prepared melatonin LNPs dispersion was cast onto a copper grid coated with carbon, and then excess liquid was removed by using filter paper. The loaded samples were allowed to dry at room temperature and photographed directly without staining. The image capture and analysis processes were carried out using the built-in software, respectively. Hence, surface morphology images obtained through TEM confirmed the spherical and monodisperse nature of our formulations (Figure.5). The findings of morphological and size analysis align with earlier reports that has shown spherical shape of melatonin loaded chitosan nanoparticles with size range 110–300 nm. [53–55]

## Differential scanning calorimetry (DSC)

**Fig. 6:**
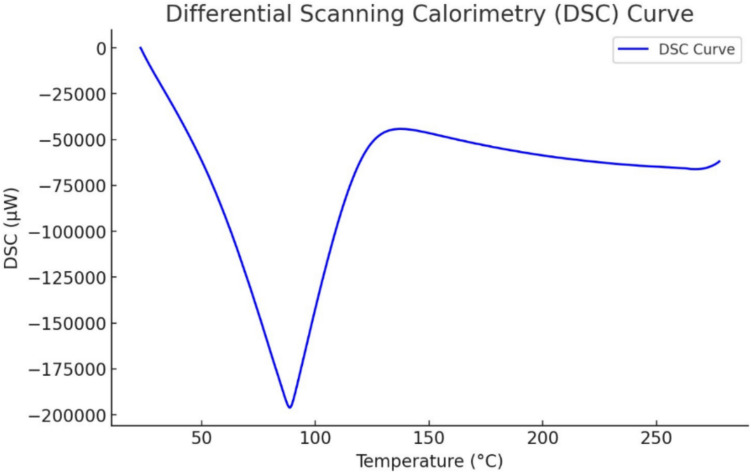
DSC Thermogram of melatonin LNPs

Thermograms of melatonin LNP were recorded utilizing a DSC thermal analyzer (Simultaneous Thermogravimetric Analyzers (STA): NEXTA STA200.Hitachi. Jaban). Each sample (16.559 mg) was heated at a temperature range of 20–300 ◦C at a rate of 10 ◦C/min in a sealed ceramic pan. The analysis was performed under a constant dry nitrogen purging rate of 20 mL/min. Simultaneously, DSC analysis was performed using empty ceramic pan as a reference standard to ensure accurate temperature calibration. (Fig. 6).

## Attenuated total reflection-Fourier transform infrared spectroscopy (ATR-FTIR)

**Fig. 7:**
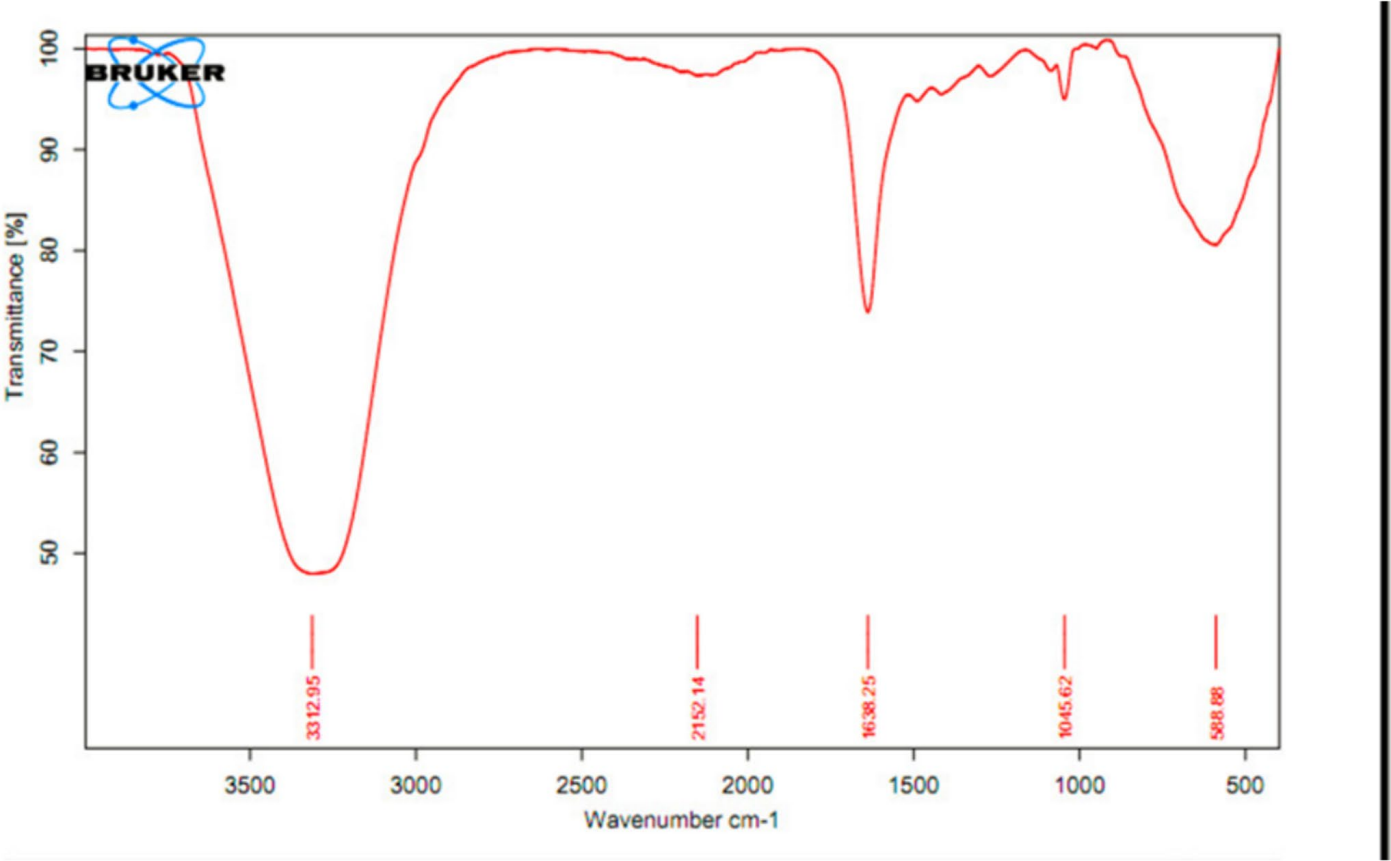
FTIR spectra of the prepared melatonin LNPs

The *Fourier transform infrared spectroscopy* of melatonin loaded LNPs were determined using ALPHA II is a compact FT-IR Spectrometer (Bruker scientific instruments Corporation, USA). The sample spectra were scanned over a range of 4000–500 wave number cm- 1.(Fig. 7).

## In vitro melatonin release from nanoparticles

### Release profile experiment

One ml of the provided formula was added to a dialysis bag (12 - 14 KD cut-off, Sigma Aldrich, Germany). The dialysis bag was sealed properly both from top and bottom and inserted into 40 ml acetate buffer pH 5.6 for 24 h in properly closed flasks. The whole system was fixed in a shaking incubator (Jeio tech SI- 300, SEOUL, KOREA) rotating at 70 RPM with temperature adjusted to 370 C. Intervals of 1, 2, 4, 6, and 24 h were set to evaluate the release of melatonin. At each interval, 1 ml sample was withdrawn from the release medium and immediately replaced with another 1 ml of warmed fresh buffer.

The withdrawn samples were measured using a UV-Spectrophotometer (JENWAY 6305, UK) at wavelength 279 nm.

## Melatonin release from nanoparticles and stability investigation

**Fig. 8:**
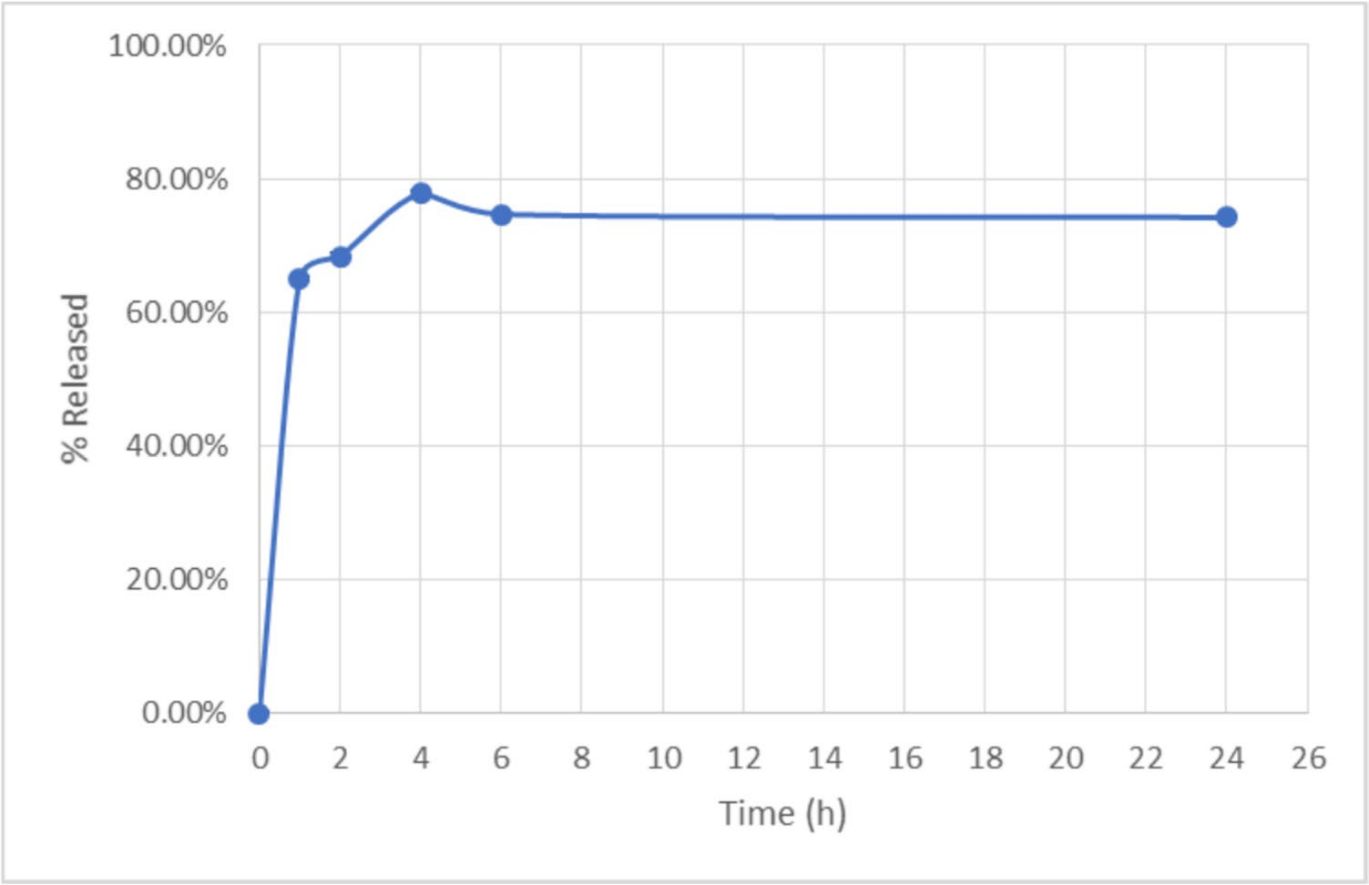
Release profile of melatonin from melatonin-loaded chitosan nanoparticles in acetate buffer (pH 5,6), 37 °C during 24 h. Data are expressed as mean ± SD (n = 5). *p < 0.05 (two-way ANOVA followed by Tukey's Multiple Comparisons test)

The melatonin release profile of the nanoparticles is shown in Fig. 8. This graph represents the cumulative percentage of drug release over time. The data suggests a rapid initial release within the first two hours, reaching approximately 70–80%. After this burst phase, the release rate stabilizes, indicating a sustained release pattern for the remaining duration up to 24 h. This profile suggests a biphasic drug release system, where an initial burst is followed by a controlled release phase. The burst release may be attributed to the surface-loaded drug, while the sustained phase likely results from gradual diffusion or polymer degradation.

It has been reported that the in vitro release of melatonin have indicated a rapid release within the first hour and similarly reached a plateau after 4 h, followed by a slowed release extending up to 8 h. [56]

## Clinical procedures

Before the start of the study, the participants were motivated by discussing the benefits of plaque control measures and the necessity of periodontal treatment. Detailed periodontal examinations and full mouth periodontal charts were obtained for the eligible participants of this study. CBCT radiographs were obtained at the predetermined sites of CAL.

Following a proper examination and diagnosis, all patients underwent a comprehensive SRP. Ultrasonic tips and Gracey curettes were used to meticulously eliminate calculus, subgingival, and supragingival plaque and the participants were instructed to apply the oral hygiene measures.

**Fig. 9:**
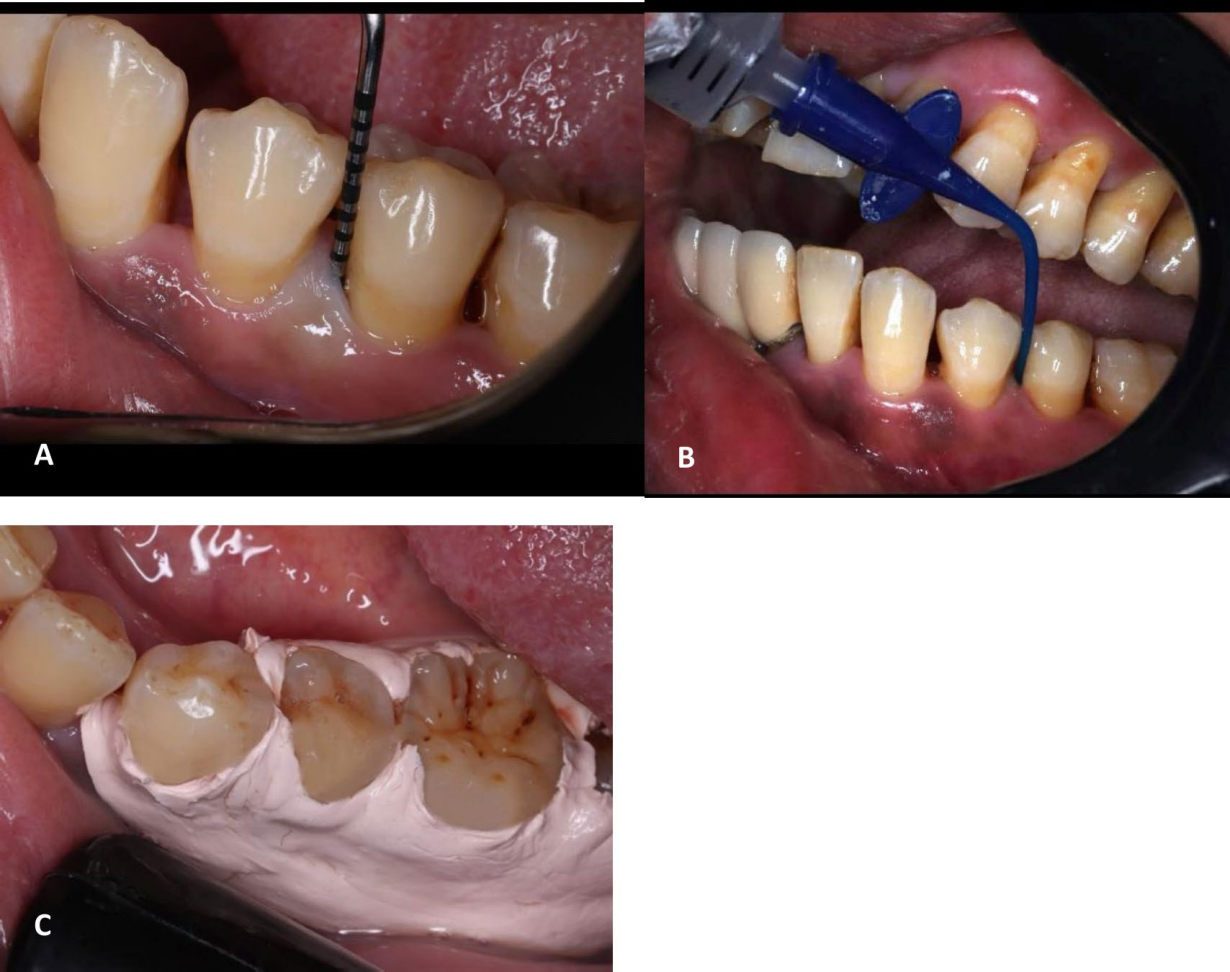
Clinical photograph showing steps of gel application: A) Assessment of PPD using graduated periodontal probe. B) Subgingival delivery of the gel using blunt nee*d*le. C)Application of periodontal pack after the LDD

After the completion of SRP, the local drug was delivered by a single operator to all patients (AA). The melatonin LNPs gel was injected in the periodontal pocket of patients of the first group, placebo gel was injected in the second group and chitosan LNPs gel was used for the third group using a syringe equipped with Endo-Eze® irrigation tips 0.014"(Ultradent Products, Inc., UT, USA). The injection was performed until the pocket was filled. For each group, the gel was applied once a week for 4 weeks. Figure 9

The gel was applied carefully without traumatizing the periodontal tissues. A periodontal pack (Coe-Pak™, GC America Inc., Alsip, IL, USA) was used to secure the area for two days following the installation of the LDD system. For one week, the participants were instructed to avoid brushing the area of gel application[46, 57], using interdental aids, or chewing sticky or hard foods. Patients were instructed for periodic recall monthly for reassurance of oral hygiene measurements then they were recalled at 3 months and 6 months postoperatively.

## Inter and intra examiner calibration:

The clinical and radiographic outcomes were evaluated at baseline and follow-up intervals. The outcomes were evaluated by two independent blind investigators (FA&SE) and the inter-examiner calibration was evaluated. We found a high agreement between both investigators (90.23%) with Cohen’s kappa coefficient (k) calculated at 0.75. In case of disagreement between the primary investigators, the third blind investigator (WS) was consulted to solve the disagreement between them. We performed the intra-examiner calibration for one clinical parameter (CAL) and one radiographic parameter (Bone defect height). A total of 17 patients were examined for calibration purposes. These patients were included in the final sample of 67 participants in the study. The measurements were calibrated before recording the baseline measurements with repeated measurements at two different points of time with intra-class correlation coefficient (ICC) for the clinical and radiographic parameters ranged from 0.85 to 0.92.

## Statistical analysis

IBM SPSS software package version 20.0. (Armonk, NY: IBM Corp) was used to analyze the data entered into the computer. Shapiro–Wilk test assessed the continuous data for normality. Quantitative data were represented as range (minimum and maximum), mean, standard deviation and median. To compare the three studied groups, One-way ANOVA test was performed, followed by Post Hoc test (Tukey) for pairwise comparison. For not normally distributed quantitative variables,the Kruskal Wallis test was utilized,followed by Post Hoc test (Dunn's for multiple comparisons test) for pairwise comparison. A repeated measures ANOVA was used for normally distributed quantitative variables, to compare between more than two time points, followed by an adjusted Bonferroni post hoc test for pairwise comparisons. To compare between two periods, paired t-test for normally distributed quantitative variables was used. Wilcoxon signed ranks test for abnormally distributed quantitative variables, to compare between two periods. The result were considered statistically significant at a 5% level (p < 0.05).

## Results

### Primary outcome variables analysis

**Fig. 10:**
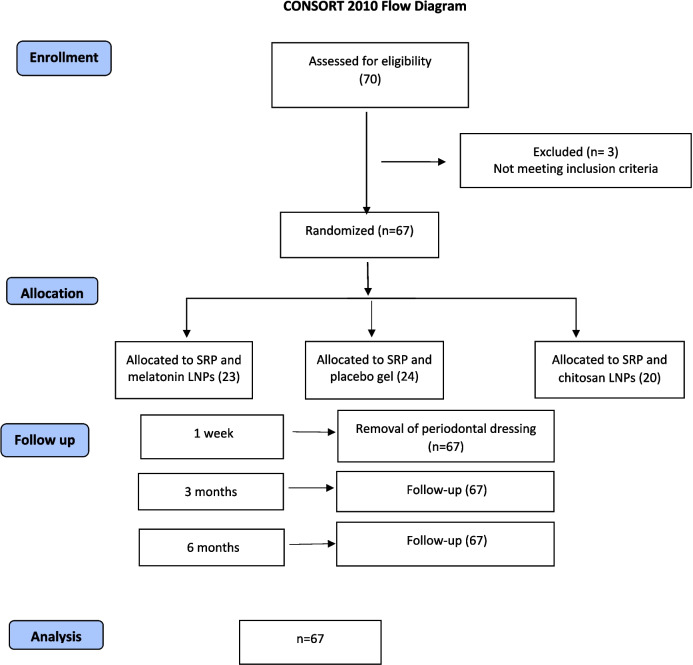
Consolidated Standards of Reporting Trials (CONSORT) flow chart for trial recruitment

After screening the potentially eligible patients (70 patients), 3 of them were excluded as they didn’t meet the eligibility criteria. The flow chart for trial recruitment is presented in Fig. 10. The current study included 67 patients with intrabony defects, 8 males and 59 females. The following clinical indices were evaluated for each patient: PI, GI, PPD, and CAL at baseline, 3 months, and 6 months, whereas radiographic parameters were evaluated at baseline and 6 months. The radiographic parameters included the height, depth, AC level, BL width, and MD width of the intrabony defects. No adverse reactions were reported during the study or side effects were observed during or after treatment with LNPs, and the gel was well tolerated by patients. Demographic characteristics of the test and the control groups are shown in Table 1. We detected non-statistically significant differences in age and gender among the studied groups.

**Table 1:** Comparison between the different groups studied according to demographic data

	Melatonin LNPs (*n* = 23)		Placebo(*n* = 24)		Chitosan LNPs (*n* = 20)		Test of Significance	*p*
	No	%	No	%	No	%		
Sex								
Male	2	8.7	1	4.2	5	25.0	FET = 5.838	0.088
Female	21	91.3	23	95.8	15	75.0		
Age (years)								
Mean ± SD	35.70 ± 10.76		38.79 ± 8.63		36.45 ± 8.59		F = 0.514	0.674

*IQR* Inter quartile range, *FET* Exact Fisher test, *F* F for One way ANOVA test, *p* *p* value

Evaluation of the PI among the three studies groups showed that PI significantly differed between groups: melatonin LNPs & placebo and melatonin LNPs & chitosan LNPs. However, there were no significant differences between groups placebo & chitosan LNPs at all the study’s evaluation times. In contrast, the assessment GI among the three study groups revealed insignificant differences at all points of time(P > 0.05). Significant differences in PPD were detected between groups melatonin LNPs &placebo (*p* < *0.001*) and melatonin LNPs & chitosan LNPs (*p* = *0.005*) at baseline while After 3 months, the difference persisted between Melatonin LNPs and Placebo (*p* < *0.001*) and between Melatonin LNPs and Chitosan LNPs (*p* = *0.027*).In addition, no differences in PPD were noticed between the groups placebo & chitosan LNPs across all measured time points, baseline, 3 months, and 6 months. Evaluation of CAL revealed significant differences between the 3 studies groups at baseline, 3 months, and 6 months.

The reduction in PPD and CAL after 6 months showed substantial variation among the three groups studied. Melatonin LNPs group exhibited the greatest reduction followed by chitosan LNPs group, while placebo group showing the least reduction.These differences were statistically significant (*p* < *0.05*), with all pairwise comparisons showing strong significance for CAL and PPD reductions. Table 2

**Table 2:** Comparison of the clinical outcomes between the three studied groups

		**Melatonin LNPs** **(n = 23)**	**Placebo** **(n = 24)**	**Chitosan LNPs** **(n = 20)**	**F**	**p**	**p**_**1**_	**p**_**2**_	**p**_**3**_
**PI**	**At baseline**	2.25 ± 0.47	2.61 ± 0.38	2.54 ± 0.32	5.434^*^	0.007^*^	0.007^*^	0.049^*^	0.820
	**After 3 months**	1.21 ± 0.40	1.50 ± 0.46	1.50 ± 0.4	3.231^*^	0.046^*^	0.028^*^	0.037^*^	0.981
	**After 6 months**	0.37 ± 0.28	0.61 ± 0.31	0.58 ± 0.22	4.925^*^	0.010^*^	0.013^*^	0.049^*^	0.916
	**Reduction after 6 m**	1.87 ± 0.36	2.0 ± 0.42	1.96 ± 0.25	0.781	0.462	> 0.05	> 0.05	> 0.05
**GI**	**At baseline**	2.32 ± 0.53	2.46 ± 0.48	2.45 ± 0.44	0.557	0.576	> 0.05	> 0.05	> 0.05
	**After 3 months**	1.15 ± 0.34	1.32 ± 0.37	1.37 ± 0.46	1.823	0.170	> 0.05	> 0.05	> 0.05
	**After 6 months**	0.37 ± 0.25	0.45 ± 0.33	0.42 ± 0.27	0.537	0.587	> 0.05	> 0.05	> 0.05
	**Reduction after 6 m**	1.96 ± 0.45	2.01 ± 0.51	2.03 ± 0.39	0.151	0.860	> 0.05	> 0.05	> 0.05
**PPD (mm)**	**At baseline**	7.20 ± 1.37	5.06 ± 1.70	5.76 ± 1.16	13.285^*^	< 0.001^*^	< 0.001^*^	0.005^*^	0.256
	**After 3 months**	6.07 ± 1.17	4.46 ± 1.66	4.97 ± 1.15	8.541^*^	< 0.001^*^	< 0.001^*^	0.027^*^	0.432
	**After 6 months**	4.90 ± 1.17	3.91 ± 1.71	4.20 ± 1.20	3.084	0.053	0.057	0.239	0.769
	**Reduction after 6 m**	2.30 ± 0.61	1.15 ± 0.37	1.56 ± 0.40	35.466^*^	< 0.001^*^	< 0.001^*^	< 0.001^*^	0.017^*^
**CAL(mm)**	**At baseline**	6.17 ± 1.29	4.73 ± 1.40	4.23 ± 1.40	12.128^*^	< 0.001^*^	0.002^*^	< 0.001^*^	0.444
	**After 3 months**	5.07 ± 1.34	4.50 ± 1.37	3.70 ± 1.39	5.372^*^	0.007^*^	0.332	0.005^*^	0.140
	**After 6 months**	3.91 ± 1.26	4.20 ± 1.40	3.20 ± 1.31	3.207^*^	0.047^*^	0.734	0.196	0.041^*^
	**Reduction after 6 m**	2.27 ± 0.42	0.53 ± 0.12	1.03 ± 0.38	167.320^*^	< 0.001^*^	< 0.001^*^	< 0.001^*^	< 0.001^*^

*F F* for One way ANOVA test, *p p* value for comparing between the studied groups. *p*_*1*_* p* value for comparing Melatonin LNPs and Placebo. *p*_*2*_* p* value for comparing between Melatonin LNPs and Chitosan LNPs,* p*_*3*_* p* value for comparing between Placebo and Chitosan LNPs.

Over the study periods, all the study groups demonstrated significant improvements in all the clinical outcomes with significant differences between baseline & 3 months, baseline & 6 months, and 3 months & 6 months (p < 0.001). Table 3

**Table 3:** Intra-group comparison of the clinical outcomes throughout the study duration

		At baseline	After		F	p	P1	P2	P3
			3 months	6 months					
PI	Melatonin LNPs	2.25 ± 0.47	1.21 ± 0.40	0.37 ± 0.28	294.649^*^	< 0.001^*^	< 0.001^*^	< 0.001^*^	< 0.001^*^
	Placebo	2.61 ± 0.38	1.50 ± 0.46	0.61 ± 0.31	300.418^*^	< 0.001^*^	< 0.001^*^	< 0.001^*^	< 0.001^*^
	Chitosan LNPs	2.54 ± 0.32	1.50 ± 0.4	0.58 ± 0.22	460.278^*^	< 0.001^*^	< 0.001^*^	< 0.001^*^	< 0.001^*^
GI	Melatonin LNPs	2.32 ± 0.53	1.15 ± 0.34	0.37 ± 0.25	265.860^*^	< 0.001^*^	< 0.001^*^	< 0.001^*^	< 0.001^*^
	Placebo	2.46 ± 0.48	1.32 ± 0.37	0.45 ± 0.33	253.506^*^	< 0.001^*^	< 0.001^*^	< 0.001^*^	< 0.001^*^
	Chitosan LNPs	2.45 ± 0.44	1.37 ± 0.46	0.42 ± 0.27	322.393^*^	< 0.001^*^	< 0.001^*^	< 0.001^*^	< 0.001^*^
PPD(mm)	Melatonin LNPs	7.20 ± 1.37	6.07 ± 1.17	4.90 ± 1.17	275.260^*^	< 0.001^*^	< 0.001^*^	< 0.001^*^	< 0.001^*^
	Placebo	5.06 ± 1.70	4.46 ± 1.66	3.91 ± 1.71	178.456^*^	< 0.001^*^	< 0.001^*^	< 0.001^*^	< 0.001^*^
	Chitosan LNPs	5.76 ± 1.16	4.97 ± 1.15	4.20 ± 1.20	241.937^*^	< 0.001^*^	< 0.001^*^	< 0.001^*^	< 0.001^*^
CAL(mm)	Melatonin LNPs	6.17 ± 1.29	5.07 ± 1.34	3.91 ± 1.26	386.926^*^	< 0.001^*^	< 0.001^*^	< 0.001^*^	< 0.001^*^
	Placebo	4.73 ± 1.40	4.50 ± 1.37	4.20 ± 1.40	98.559^*^	< 0.001^*^	< 0.001^*^	< 0.001^*^	< 0.001^*^
	Chitosan LNPs	4.23 ± 1.40	3.70 ± 1.39	3.20 ± 1.31	99.750^*^	< 0.001^*^	< 0.001^*^	< 0.001^*^	< 0.001^*^

*F: F* test (ANOVA) with repeated measures, the significant difference between periods was done using Post Hoc Test (adjusted Bonferroni), *p p*-value for comparing between the three studied periods, *p*_*1*_* p-*value for comparing between at baseline and after 3 months in each group, *p*_*2*_* p*-value for comparing between at baseline and after 6 months in each group, *p*_*3*_* p*-value for comparing after 3 months and after 6 months in each group.

### Radiographic Parameters of Intraosseous Defects on CBCT

**Fig. 11:**
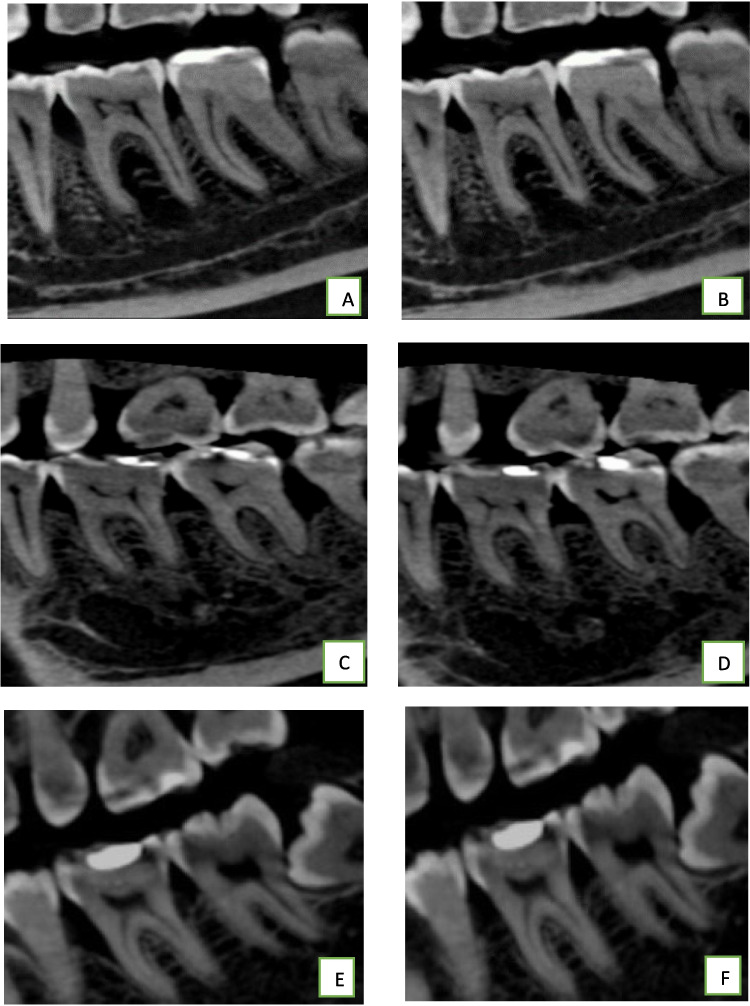
showing the comparison of sagittal views of the intrabony defects in CBCT at baseline and after 6 months. (**A**) Intraosseous defect on the mesial surface of the lower first molar at the melatonin LNPs group at baseline, (**B**) Reduction in the depth of the intraosseous defect in the melatonin LNPs group after 6 months, (**C**) Intraosseous defect on the distal surface of lower second molar at the placebo group at baseline. (**D**) No change in the intraosseous defect in the sagittal view in placebo group after 6 months, (**E**) Intraosseous defect on mesial surface of lower first molar at the chitosan LNPs group at baseline, and (**F**) Reduction in the intraosseous defect depth in the chitosan LNPs group after 6 months.

Evaluation of the height of intrabony defect showed that there were significant changes in the reduction of the height after 6 months between all the study groups while melatonin LNPs group demonstrated the highest significant reduction after 6 months (2.10 ± 0.34, P < 0.001) with a smaller but significant change in chitosan LNPs group (0.56 ± 0.09, P < 0.001). However, the Placebo group showed minimal insignificant change in the height of the intrabony defect (0.05 ± 0.19, p = 0.153). Table 4 & Fig. 11.

**Table 4:** Comparison of the radiographic outcomes among the three studied groups

		**Melatonin LNPs** **(n = 23)**	**Placebo** **(n = 24)**	**Chitosan LNPs** **(n = 20)**	**Test of Significance**	**p**	**p1**	**p2**	**p3**
**Height(mm)**	**At baseline**	6.31 ± 1.29	4.86 ± 1.53	4.24 ± 1.55	F = 14.100^*^	< 0.001^*^	0.001^*^	< 0.001^*^	0.308
	**After 6 months**	4.21 ± 1.22	4.80 ± 1.53	3.68 ± 1.52	F = 3.898	0.024^*^	0.245	0.396	0.019^*^
	**Reduction**	2.10 ± 0.34	0.05 ± 0.19	0.56 ± 0.09	F = 559.817^*^	< 0.001^*^	< 0.001^*^	< 0.001^*^	< 0.001^*^
	**p4**	< 0.001^*^	0.153	< 0.001^*^					
**Depth(mm)**	**At baseline**	2.59(	1.83(0.85–3.56)	1.92(0.85–4.55)	H = 13.016^*^	0.001^*^	< 0.001^*^	0.016^*^	0.438
	**After 6 months**	0.85(0.02–3.93)	1.82(0.84–3.56)	1.56(0.50–4.16)	H = 16.052^*^	< 0.001^*^	< 0.001^*^	0.009^*^	0.340
	**Reduction**	1.62(1.33–2.31)	0.01(− 0.24–0.42)	0.33(− 1.17–1.89)	H = 48.777	< 0.001^*^	< 0.001^*^	< 0.001^*^	0.299
	**p4**	< 0.001^*^	0.053	0.063					
**Level of AC(mm)**	**At baseline**	3.42(1.54–6.26)	3.60(1.32––6.10)	1.75(0.72–4.11)	H = 15.162^*^	0.001^*^	0.825	0.001^*^	< 0.001^*^
	**After 6 months**	2.70(0.87–5.54)	3.64(1.35–6.14)	1.56(0.56–3.97)	H = 16.843^*^	< 0.001^*^	0.033^*^	0.029^*^	< 0.001^*^
	**Reduction**	0.67(0.51–1.19)	− 0.03(− 0.50–0.45)	0.18(− 0.51–0.80)	H = 46.339^*^	< 0.001^*^	< 0.001^*^	< 0.001^*^	0.024^*^
	**p4**	< 0.001^*^	0.103	0.054					
**BL width(mm)**	**At baseline**	6.54 ± 1.71	5.35 ± 1.56	5.87 ± 1.14	F = 4.474^*^	0.015^*^	0.011^*^	0.274	0.274
	**After 6 months**	4.43 ± 1.62	5.31 ± 1.56	5.29 ± 1.12	F = 3.190^*^	0.047^*^	0.027^*^	0.046^*^	0.967
	**Reduction**	2.11 ± 0.51	0.05 ± 0.13	0.58 ± 0.06	F = 316.843^*^	< 0.001^*^	< 0.001^*^	< 0.001^*^	< 0.001^*^
	**p4**	< 0.001^*^	0.061	< 0.001^*^					
**MD width(mm)**	**At baseline**	2.91 ± 0.71	2.36 ± 0.66	2.22 ± 0.58	F = 8.206^*^	0.001^*^	0.006^*^	0.001^*^	0.744
	**After 6 months**	1.55 ± 0.72	2.32 ± 0.67	1.84 ± 0.59	F = 9.683^*^	< 0.001^*^	< 0.001^*^	0.281	0.043^*^
	**Reduction**	1.36 ± 0.20	0.05 ± 0.14	0.38 ± 0.08	F = 564.480^*^	< 0.001^*^	< 0.001^*^	< 0.001^*^	< 0.001^*^
	**p4**	< 0.001^*^	0.092	< 0.001^*^					

Normally quantitative data was expressed in Mean ± SD. While non-normally distributed data was expressed in Median (Min. – Max.), *H H* for Kruskal Wallis test, Post Hoc Test (Dunn's for multiple comparisons test) was used for pairwise comparison between each two groups, *F F* for One way ANOVA test, pairwise comparison between each 2 groups was done using Post Hoc Test (Tukey), *p p* value for comparison between the three studied groups, *p*_*1*_* p* value for comparing Melatonin LNPs and Placebo, *p*_*2*_* p* value for comparing between Melatonin LNPs and Chitosan LNPs, *p*_*3*_* p *value for comparing between Placebo and Chitosan LNPs, *p*_*4*_* p *value for comparing between baseline and after 6 months in each group.

Regarding the defect depth at 6 months, we observed statistically significant differences between melatonin LNPs and placebo group as well as melatonin LNPs and chitosan LNPs group. However, there is no statistically significant difference between placebo and chitosan LNP group. In addition, the melatonin group demonstrated the highest significant reduction in defect depth after 6 months (P < 0.001). However, Placebo and Chitosan LNPs groups showed insignificant changes in the depth of the intrabony defect (P > 0.05).

The change in the level of AC was evaluated at baseline and after 6 months. We observed a significant change in the level of AC in the melatonin LNPs group at 6 months, with slight changes in the placebo and chitosan LNPs groups. The most pronounced change from baseline was in melatonin LNPs group (P < 0.001) with insignificant changes in placebo and chitosan LNPs group (P > 0.05).

Evaluation of the BL and MD width of intrabony defect showed that there were significant changes in both measurements after 6 months between the three groups (P < 0.001). Melatonin LNPs demonstrated the highest significant reduction after 6 months (P < 0.001), with a smaller but significant change in chitosan LNPs group. However, the Placebo group showed minimal insignificant changes in the BL and MD width of intrabony defect. Table 4

## Discussion

The scarcity of studies investigating the effect of melatonin LNPs as a novel local drug delivery in the treatment of periodontitis highlights an important gap in periodontal research. Hence, we conducted the first triple-blind, randomized, placebo-controlled clinical trial to evaluate the effects of locally applied melatonin LNPs as an adjunct to non-surgical periodontal treatment in patients with intrabony defects. The current study's findings highlight the positive effect of local delivery of melatonin LNPs in the treatment of periodontitis, as evidenced by the positive impact seen in the radiographic and clinical outcomes.

Comparison of the clinical and radiographic outcomes in the melatonin LNPs group showed significant differences between baseline and follow-up points of time. Additionally, compared to the control groups, melatonin LNPs-treated sites demonstrated a more statistically significant reduction in PPD and a more statistically significant change in CAL. These findings support the research by Ahmed et al. [58] and Montero et al. [59], which found that topical melatonin application has several beneficial effects on periodontal health and significantly improves clinical parameters like pocket depth and GI. In addition, topical melatonin application improved bone formation and osteoblast differentiation as reported by Gonde et al.[57]’ clinical study In which they applied 1% melatonin gel as LDD for patients with intrabony defects and follow the cases for 6-months.

Melatonin has been shown to have significant antioxidant, anti-inflammatory, and immunomodulatory properties in addition to its potent endogenous action as a free-radical scavenger[60], making it a key molecule for periodontal protection. This may be the reason for the reported improvements in PPD and CAL. By preventing periodontal damage, it also plays a vital role in maintaining periodontal homeostasis. [61, 62]

Comparing the reduction of PI and GI at 6 months revealed insignificant variations between the groups. These results may be attributed to the fact that these indices reflect the oral hygiene practice and the amount of inflammation among the participants regardless of the type of intervention making results similar across groups. In addition, all the study’s participants received periodontal debridement with oral hygiene instructions. So, PI and GI may not act as sensitive indices to measure major differences between the different treatment modalities used. [63]

The current clinical trial revealed a substantial improvement in the radiographic parameters with a more remarkable amount of bone fill on CBCT after 6 months when melatonin LNPs gel was employed as an adjuvant to SRP for the treatment of intraosseous defects. In addition, we detected a higher amount of bone fill in the melatonin LNPs group than chitosan LNPs and placebo groups. These findings agree with a study that used melatonin gel as an LDD system for intrabony defects, At the end of six months, our study revealed a significant decrease in the intrabony depth of defect and a considerable vertical defect fill. [57] In addition, Shoukheba et al. conducted a histological analysis of beagle dogs. In which the melatonin group showed new attachment without any epithelial downgrowth, and the entire periodontal bone defect was filled with narrow marrow spaces and new lamellar bone [64].

Several factors can explain the bone fill in the melatonin LNPs group. First, melatonin possesses several biological effects on bone through modulation of bone production and resorption. [65] Melatonin first enhances the growth of pre-osteoblasts and osteoblast-like cells, stimulates the creation of mineralized matrix, and raises the expression of type I collagen and bone marker proteins including alkaline phosphatase, osteopontin, bone sialoprotein, and osteocalcin. [66–68]

Melatonin can stimulate the Osteoblasts [69] through stimulating the BMP/ERK/Wnt signaling pathways, which in turn increases osteoblast growth [70]. Another study found that melatonin receptors allow melatonin to induce the differentiation of human adult mesenchymal stem cells into mature osteoblasts [66]. Melatonin inhibits the formation of osteoclasts by promoting the expression of osteoprotegerin in osteoblasts, a substance considered to be antagonistic for RANKL [71]. Melatonin inhibits the RANKL-induced nuclear factor kappa B (NF-kB) pathway, which reduces osteoclast formation [72].

In addition, melatonin can indirectly control bone metabolism through interacting with other molecules or systemic hormones such as parathyroid hormone, calcitonin, and estrogen. Ladizesky et al. [73] demonstrated that in ovariectomized rats, estradiol administration may prolong the impact of melatonin to accelerate bone remodeling. Moreover, melatonin can enhance the elimination of the free radicals produced by osteoclasts during bone resorption and protect bone cells from oxidative stress [21, 74]. It can act as a significant modulator of calcium metabolism enhancing the prevention of osteoporosis and hypocalcemiaIncreased trabecular density and bone-implant contact were observed when melatonin was applied locally around dental implants [75]. Furthermore, melatonin increases angiogenesis and preserves capillary homeostasis during bone defect healing. [76] It can penetrate the cell membrane and reach subcellular component and nucleus. [77–79]

After 6 months, we detected a change in the alveolar crest's level as indicated by a change in the CEJ-AC distance. Melatonin LNPs and chitosan LNPs groups showed a decrease in CEJ-AC distance while placebo group showed an increase in the CEJ-AC distance. This suggests that the placebo group had a greater rate of alveolar crest resorption.

Multiple systems demonstrated improved accuracy and sustained delivery of melatonin when it was integrated into various nanocarriers. Melatonin-loaded LNPs demonstrated better antioxidant, anti-inflammatory, and anticancer activities in many cell types and biological tissues than melatonin in its free form [80]. The nanoparticle delivery system utilized in the current study possessed a synergetic effect with melatonin. They created a localized, sustained, and biologically conducive environment for periodontal regeneration. The nanoparticles enhanced the sustained release of melatonin at the bone defect, improving its efficacy and decreasing its systemic effect. The LNPs are engineered to adhere to application location as in periodontal tissues that maximize the effect of the melatonin at the site of application. In addition, encapsulation of melatonin by nanoparticles decreases the degradation liability of melatonin and increases its bioavailability. [29, 81, 82]

Some nanoparticle carriers such as hydroxyapatite and chitosan possess osteoinductive properties. In the melatonin LNPs group, the nanocarrier is the chitosan, which is a biocompatible carrier with a potential osteoinductive criteria. Hence, we conducted a separate group for the chitosan LNPs to distinguish the individual effect of melatonin LNPs from the effect of chitosan LNPs in bone formation [83, 84].

In the chitosan LNPs group, we found a significant improvement in the radiographic measurements of the bone defect. In a previous In vivo study, it was suggested that chitosan is able to stimulate osteogenesis alone. [84] In addition, the stimulation of trabecular bone production by chitosan nanofiber scaffold enhances bone healing. [83] Another study measured the efficacy of chitosan nanohydrogel as a bone regenerative agent and the authors concluded that that chitosan may be utilized in bone repair. [85] Moreover, Mathews et al. reported that chitosan enhanced the mineralization during differentiation of osteoblasts by upregulating the associated genes. [86] Chitosan supports the attachment as well as proliferation of osteoblasts and the formation of mineralized bone matrix [87]. In addition, chitosan showed an osteoconductive property in surgically created bone defects [88]. In addition to being used as porous scaffolds that mimic the extracellular matrix of bone, providing a structure for new bone cells to grow within [89], chitosan was known for its biocompatibility, biodegradability, and antimicrobial activity [90].

The chitosan’s surface is positively charged and it has a permeation-enhancing action that can interact with the cell membrane and enhance mucosal absorption by bypassing epithelial barriers [91]. Additionally, research has shown that chitosan LNPs can open cellular tight junctions by reorganizing the structure of proteins linked to tight junctions. [92] Chitosan is a good choice for delivering genes and medications because of its many other characteristics, including bio-adhesiveness, low toxicity, permeability, and pharmaceutical features [93]. It exhibits mucoadhesive qualities and offers adherence to a mucosal surface which allows improved and prolonged drug adsorption [94].

In our study, we noticed significant differences in PPD, CAL, and the height, depth, BL width, and MD width of intra-bony defects at baseline between the 3 groups. To exclude the effect of baseline variability on the treatment, we relied on the amount of reduction rather than absolute baseline values. Also, reduction reflects actual improvement in each group, indicating the success of the treatment. So, the observed significant reduction in PPD and CAL in the Melatonin LNPs suggests that the treatment used gives a superior regenerative effect and this effect is independent of baseline values. In addition, this is a randomized controlled clinical trial, so all patients regardless of the defect morphology have an equal chance to be enrolled in any group. So, randomization reduces bias. The treatment outcome is influenced by the morphology of intra-bony defect. A systematic review by Pelekos and colleagues concluded that deeper defects with narrower angles and a greater number of walls showing more favorable results in CAL gain and bone gain regardless of the biomaterials used. This suggests that treatment outcomes depend on the defect morphology.[95] However, not all studies found a relationship between bone morphology and healing outcomes. For example, a pilot exploratory analysis by Nibali et al. reported no significant relationship between defect depth, angle, or predicted number of walls and clinical or radiographic healing 12 months after minimally invasive non-surgical therapy which suggests that treatment outcomes may depend on factors other than morphology [96].

In conclusion, the current study revealed that melatonin and chitosan LNPs can act as novel and effective adjunctive local drug therapies in the management of periodontal intrabony defects. We found significant improvements in the clinical and radiographic outcomes when melatonin and chitosan LNPs were combined with non-surgical periodontal therapy. In addition to its immunomodulatory, anti-inflammatory, and antioxidant properties of melatonin, the current study showed that it can enhance bone formation. So, it could potentially eliminate the need for surgical interventions in the management of periodontal diseases.

## Limitations

Even with the promising effects of melatonin LNPs LDD in improving the clinical and radiographic outcomes of periodontal intrabony defects, the current study challenged certain limitations. First, we recommend future larger sample sized clinical trials to improve the generalizability of our results. Second, we recommend longer follow up durations up to five years to measure the long-term effects of using melatonin and chitosan LNPs on the clinical and radiographic outcomes and to understand their mechanisms of action and safety.

## Data Availability

The data that support the findings of this study are available from the corresponding author upon reasonable request.
